# OP48 Establishing The Implant Subsidy List In Singapore: A Collaborative Approach Between Health Technology Assessment And National Procurement Agencies

**DOI:** 10.1017/S0266462324001107

**Published:** 2025-01-07

**Authors:** Yen Ee Wong, Wayne Ding, Yan Teck Ho, Swee Sung Soon, Kwong Ng

## Abstract

**Introduction:**

Public healthcare institutions (PHIs) experience rising and discordant implant costs. Implementing subsidies for implants without concurrent price harmonization through national procurement contracts impedes equitable patient access. This abstract describes the collaboration between the Agency for Care Effectiveness (ACE), Singapore’s national health technology assessment (HTA) agency, and the public healthcare supply chain agency (ALPS) to establish the Implant Subsidy List (ISL).

**Methods:**

The alignment between ISL and concurrent national procurement contracts for implants required close coordination and co-development of streamlined and scalable processes. To achieve this, ACE and ALPS collaborated to understand the complex implant landscape by leveraging on known vendors and products and pre-empting upcoming ones, identifying key opinion leaders, and sharing technical expertise. Both agencies also developed common-use templates and streamlined vendor submission processes. Of note, joint request-for-proposal (RFP) exercises for national subsidy and procurement listings combined negotiation efforts to achieve lower implant prices. Regular cross-agency discussions enabled continuous knowledge sharing and process refinement in an evolving implants landscape.

**Results:**

For phase one rollout of ISL, 15 implant topics across eight clinical functions were evaluated, supported by concurrent establishment of 15 national procurement contracts. As of December 2023, ACE listed 22,689 ISL implants for implementation, with more topics onboarded on a rolling basis. ACE and ALPS streamlined process flows, standardized communications channels and information, and utilized contractual tools. Vendors could submit for both subsidy and procurement through the secure ALPS portal, reducing duplication of work and errors while allowing procurement and subsidy consideration processes to proceed concurrently. Combined negotiation efforts have resulted in an average price reduction of 19 percent.

**Conclusions:**

The alignment between national HTA and procurement agencies is important for successful scaling. The ACE–ALPS collaboration has synergized both agencies’ understanding of the complex implant landscape and established streamlined workplans and processes to support initial establishment of ISL and its maintenance. Challenges include navigating strict procurement rules and continuous cross-agency improvement efforts to ensure applicability in an evolving landscape.

